# Comparative analysis of cellular expression pattern of schizophrenia risk genes in human versus mouse cortex

**DOI:** 10.1186/s13578-019-0352-5

**Published:** 2019-11-04

**Authors:** Hai-Long Zhang, Jia-Wen Long, Wei Han, Jiuzhou Wang, Weichen Song, Guan Ning Lin, Dong-Min Yin

**Affiliations:** 1 Key Laboratory of Brain Functional Genomics, Ministry of Education and Shanghai, School of Life Science, East China Normal University, Shanghai, 200062 China; 2 Department of Mathematics, Southern University of Science and Technology, Shenzhen, China; 3 Shanghai Key Laboratory of Psychotic Disorders, Shanghai Mental Health Center, Shanghai Jiao Tong University School of Medicine, Shanghai, China; 4 School of Biomedical Engineering, Shanghai Jiao Tong University, Shanghai, China

**Keywords:** Schizophrenia, Gene, Single-cell RNA sequence, Cortex, Human and mouse, Glutamatergic neuron, GABAergic neuron, Non-neuronal cell

## Abstract

**Background:**

Schizophrenia is a common psychiatric disease with high hereditary. The identification of schizophrenia risk genes (SRG) has shed light on its pathophysiological mechanisms. Mouse genetic models have been widely used to study the function of SRG in the brain with a cell type specific fashion. However, whether the cellular expression pattern of SRG is conserved between human and mouse brain is not thoroughly studied.

**Results:**

We analyzed the single-cell transcription of 180 SRG from human and mouse primary visual cortex (V1). We compared the percentage of glutamatergic, GABAergic and non-neuronal cells that express each SRG between mouse and human V1 cortex. Thirty percent (54/180) of SRG had significantly different expression rate in glutamatergic neurons between mouse and human V1 cortex. By contrast, only 5.6% (10/180) of SRG showed significantly different expression in GABAergic neurons, which is similar with the ratio of SRG (15/180) with species difference in total cell populations. Strikingly, the percentage of non-neuronal cells expressing all SRG are indistinguishable between human and mouse V1 cortex. We further analyzed the biological significance of differentially expressed SRG by gene ontology. The species-different SRG in glutamatergic neurons are highly expressed in dendrite and axon. They are enriched in the biological process of response to stimulus. However, the differentially expressed SRG in GABAergic neurons are enriched in the regulation of organelle organization.

**Conclusion:**

GABAergic neurons are more conserved in the expression of SRG than glutamatergic neurons while the non-neuronal cells show the species conservation for the expression of all SRG. It should be cautious to use mouse models to study those SRG which show different cellular expression pattern between human and mouse cortex.

## Background

Schizophrenia (SZ) is a severe, disabling mental illness affecting about 1% of population [1]. It is estimated that the heritability of SZ is about 0.8 [2], which indicate the substantial genetic contribution to the disease. To illustrate the complex genetic etiology, large amount of genetic studies, both genome-wide and small scale, have been conducted on SZ [2]. Although hypothesis-free, genome-wide studies are capable of discovering schizophrenia risk genes (SRG) [3], it is necessary to validate the genetic results through functional studies [4]. To this end, mouse models are frequently used to study biological function and pathological consequence of SRG [5]. The convenience and accessibility of mouse models (transgenic, knock in/out, optogenetics, etc.) promoted to understand the function of SRG in the brain at the cellular and circuit levels.

Using mouse models to study SZ have been challenged due to the species difference [6]. On one hand, human brain may be unique for some high-level functions which are affected in SZ (for example, cognition, decision, etc.) [7]. Efforts have been made to evaluate and decrease the difference between human and psychiatric mouse model in terms of behavioral assessment and pharmacology [8, 9]. On the other hand, the cellular expression pattern of SRG may have species difference in human versus mouse brain. However, a comprehensive assessment of species difference of SRG in cortex of human and mouse is still lacking.

Assessing species difference of brain genetic architecture is a complex multi-dimensional task [10]. In this study, we managed to evaluate the species difference from the aspect of gene expression profile. We assume that, mouse models could properly reflect the function of a gene only if this gene has similar expression profile in mouse and human brain; or more specifically, percentage of cells that express this gene should be similar in human and mouse. Based on this hypothesis, we curated an SRG list with 180 genes and compared their expression profile in three cell types (glutamatergic, GABAergic and non-neuronal cell) from human and mouse cortex. The results from this study would allow us to evaluate the reliability of mouse models to study the function of SRG in the brain.

## Methods

### Schizophrenia risk gene list

We included two types of SRG into our list: (1) Data-driven SRG inferred from PGC GWAS [11]. SNP annotation and disease genes filtration were accomplished by Lin et al. [12]. A total of 132 SRG were identified. (2) Literature-curated SRG from multiple database [13]. Most of these SRG were identified by previous candidate gene studies [14–21]. A total of 54 literature-oriented SRG were identified. We removed overlapped genes and genes without mouse homolog from the total gene list. 180 SRG were obtained for further analysis.

### Single cell transcriptome of human and mouse brain

Single cell RNA-seq data were obtained from Allen Institute [22] (https://celltypes.brain-map.org/download#transcriptomics). Following data were chosen for analysis: (1) Single cell transcriptome of adult human primary visual cortex (V1) with 8988 nuclei. (2) Single cell transcriptome of adult mouse V1 with 15,413 cells.

### Characterization of cell type

Cell type characterization is based on the identification of reference datasets and the specific expression of marker genes. First, we calculate the Spearman correlation value between the expression profile of each cell to be identified and the expression profile of each cell annotated in the reference data set by SingleR software package. Then, according to the correlation score, we select the cell type which has the greatest correlation with the expression profile of the cell to be identified in the data set as the final cell type. The reference data set used in this project is Human Cell Landscape: a total of 1300 cell types collected by Guo Guoji’s team (http://bis.zju.edu.cn/HCL/index.html). The final annotation results of cell type were obtained according to the specific expression distribution of marker genes of known cell types (feature plot) and the identification results of data sets. Cell types were summarized to three main types: glutamatergic neuron, GABAergic neuron and non-neuronal cells.

### Statistical analysis of expression profile

We defined that a gene e was expressed in a cell when > 0 of reads from a cell were aligned to e. For each cell type and each SRG e, we counted the number of cells for each type that expressed or did not expressed gene e and put the numbers in the following table.

**Table:** 

	Human cortex	Mouse cortex	Total
Expressed	a	b	a + b
Non-expressed	c	d	c + d
Total	a + c	b + d	a + b + c + d

Whether the percentage of cells for each type that express SRG e was significantly different between human and mouse cortex was determined by χ^2^ test:

$$\chi^{2} = \mathop \sum \limits_{{\left\{ {a,b,c,d} \right\}}} \frac{{\left( {a - a_{e} } \right)2}}{a}$$

where $a_{e}$ represents expected frequency of grid a:

$$a_{e} = \frac{{\left( {a + c} \right)\left( {a + b} \right)}}{a + b + c + d}$$

Calculated χ^2^ values were adjusted for multiple testing by 180 × 3 = 540. SRG with *p* value smaller than 0.05 were considered as species difference. All analysis was conducted using *chisq. test* R function.

### Biological significance of gene sets

To test whether different gene sets obtained from previous analysis are enriched in any biological pathways, we applied Gene Ontology [23] enrichment analysis by DAVID online tool [24]. Biological Process (GO-BP), Cell Component (GO-CC) and Molecular Function (GO-MF) were analyzed. Enrichment analysis was achieved by hypergeometric test. Suppose n is the size of tested gene set s, K is the total number of genes in a biological pathway P, N is the total number of background genes (genes with GO annotation). If we randomly selected n genes from background N, we expected to select k genes from pathway p with a probability $P_{r} \left( {x = k} \right)$ that follows a hypergeometric distribution.

$$P_{r} \left( {x = k} \right) = \frac{{\left( {\begin{array}{*{20}c} K \\ k \\ \end{array} } \right)\left( {\begin{array}{*{20}c} {N - K} \\ {n - k} \\ \end{array} } \right)}}{{\left( {\begin{array}{*{20}c} N \\ n \\ \end{array} } \right)}}$$

If $k$ is large enough such that $p = P_{r} \left( {x \ge k} \right)$ is small, we could draw the conclusion that tested gene set s is enriched in pathway P. p-value for hypergeometric tests were adjusted for multiple testing by Benjamin-Hochberg method. Pathways (GO-BP, CC or MF) with adjusted p < 0.05 were considered enriched pathways.

## Results

### Cell type calls from the human and mouse primary visual cortex (V1)

**Fig. 1:**
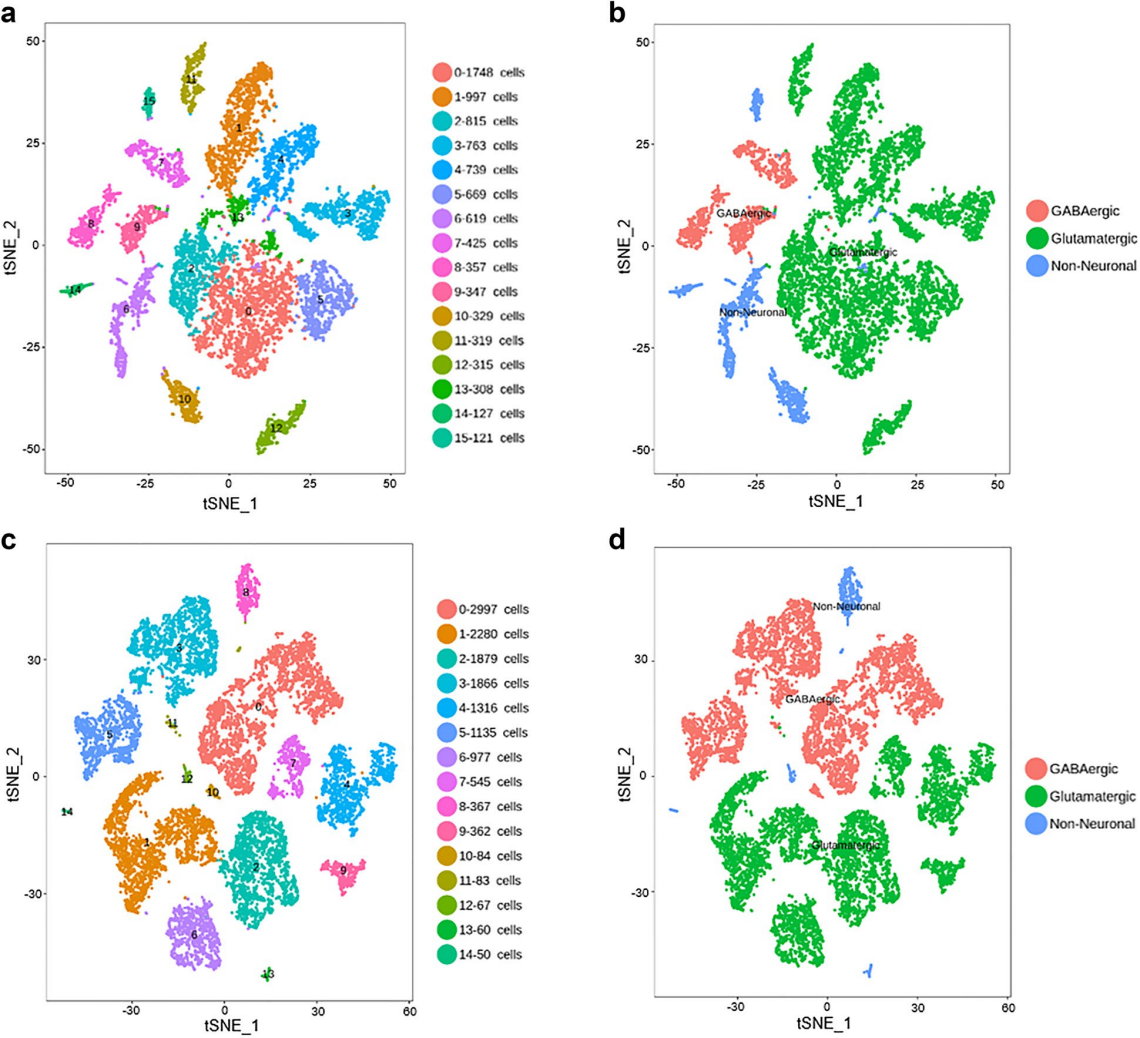
Cell-type taxonomy in human and mouse V1 cortex. **a**, **b** t-distributed stochastic neighbor embedding (tSNE) visualization of 8998 nuclei from human V1 cortex grouped by expression similarity and colored by cluster (**a**) and cell type (**b**). **c**, **d** tSNE visualization of 15,413 cells from mouse V1 cortex grouped by expression similarity and colored by cluster (**c**) and cell type (**d**). 14,048 out of 15,413 cells can be grouped into three major cell types

We sought to compare the cellular expression pattern of SRG from matched brain regions in human versus mouse. We focused on the primary visual cortex (V1) because V1 is the only brain region where the single cell RNA-seq data is currently available for both human and mouse cortex in Allen Brain Institute. Analysis of the single cell RNA-seq data of human V1 cortex reveals approximately 16 transcriptionally distinct cell types, subdivided into 3 GABAergic neuron types, 9 glutamatergic neuron types and 4 non-neuronal cell types (Fig. 1a, b). Assay of the RNA-Seq data from mouse V1 cortex reveals 15 transcriptomic cell types, divided into 4 GABAergic neuron types, 7 glutamatergic neuron types and 4 non-neuronal cell types (Fig. 1c, d). The number of SRG-positive and SRG-negative cells in the population of glutamatergic neuron, GABAergic neuron and non-neuronal cell were listed in Additional file 1: Table S1.

### Expression of SRG in total cell population from human versus mouse V1 cortex

**Fig. 2:**
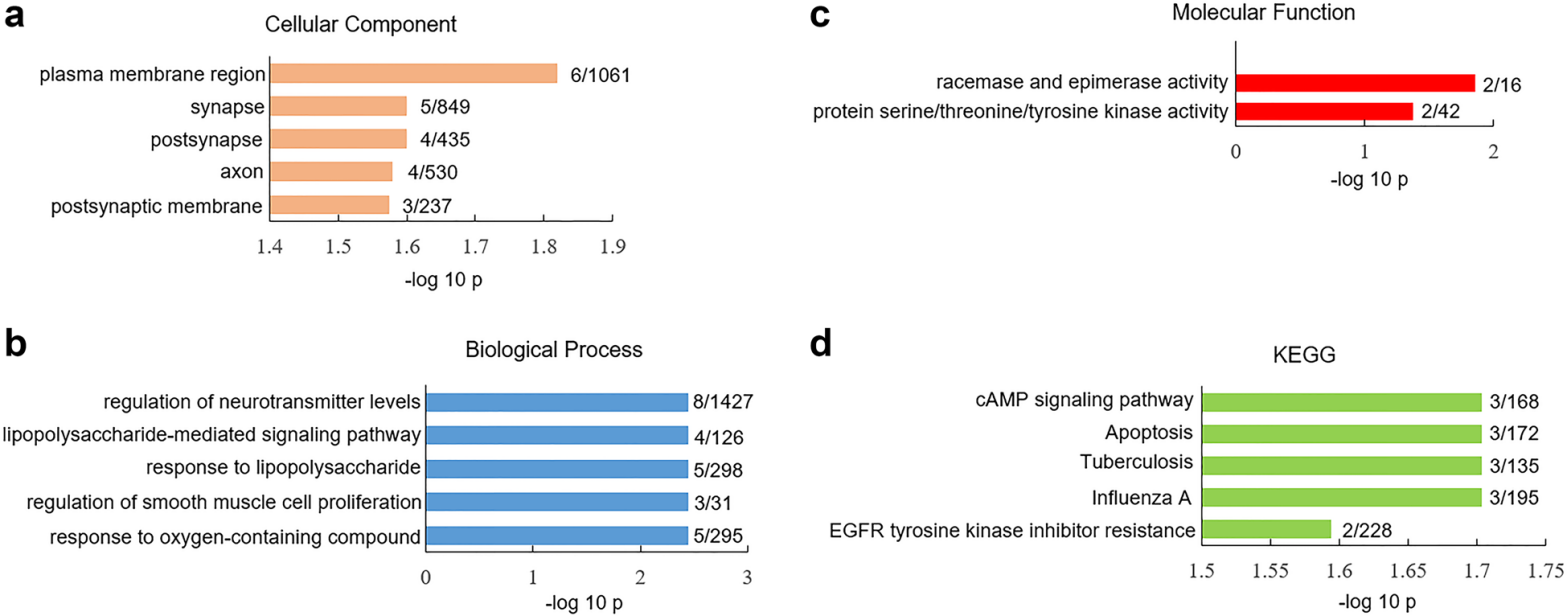
GO and KEGG analysis of differential expressed SRG in total cell population between human versus mouse V1 cortex. Significantly overrepresented cellular component (**a**), biological process (**b**) and molecular function (**c**). **d** Significantly overrepresented KEGG. The x-axis represents the value of −log_10_p, the y-axis indicates the item of GO or KEGG, the numbers after each bar indicate the list hits/pop hits

In the human and mouse V1 cortex, a total of 8998 and 15,413 cells were RNA-sequenced, respectively. The percentage of cells that expressed SRG was determined by the ratio of SRG-positive cells to total cells (Additional file 2: Table S2). The percentages of cells expressing most SRG (165 out of 180) were similar between human and mouse V1 cortex (Additional file 2: Table S2). The percentage of cells that express Akt1, Amacr, Btg1, CD34, Comt, Dtnbp1, IL18, Lsm1, Mapk3, Mcl1, Ptn, Sigmar1, Slc1a1, Srr, Vipr2 was significantly lower in human V1 cortex than mouse V1 cortex (Additional file 2: Table S2). We did gene ontology (GO) assay for these 15 SRG with species difference: they were highly expressed in the plasma membrane and synaptic region (Fig. 2a) and were overrepresented in biological processes such as regulation of neurotransmitter levels (Fig. 2b). The analysis of molecular function (MF) indicated that these 15 SRG were significantly presented in racemase (p = 1.41 × 10^−2^) and kinase activity (p = 4.3 × 10^−2^) (Fig. 2c). KEGG assay showed that these 15 SRG were enriched in cAMP signaling pathway (p = 1.98 × 10^−2^) (Fig. 2d).

### Expression of SRG in glutamatergic neurons from human versus mouse V1 cortex

**Fig. 3:**
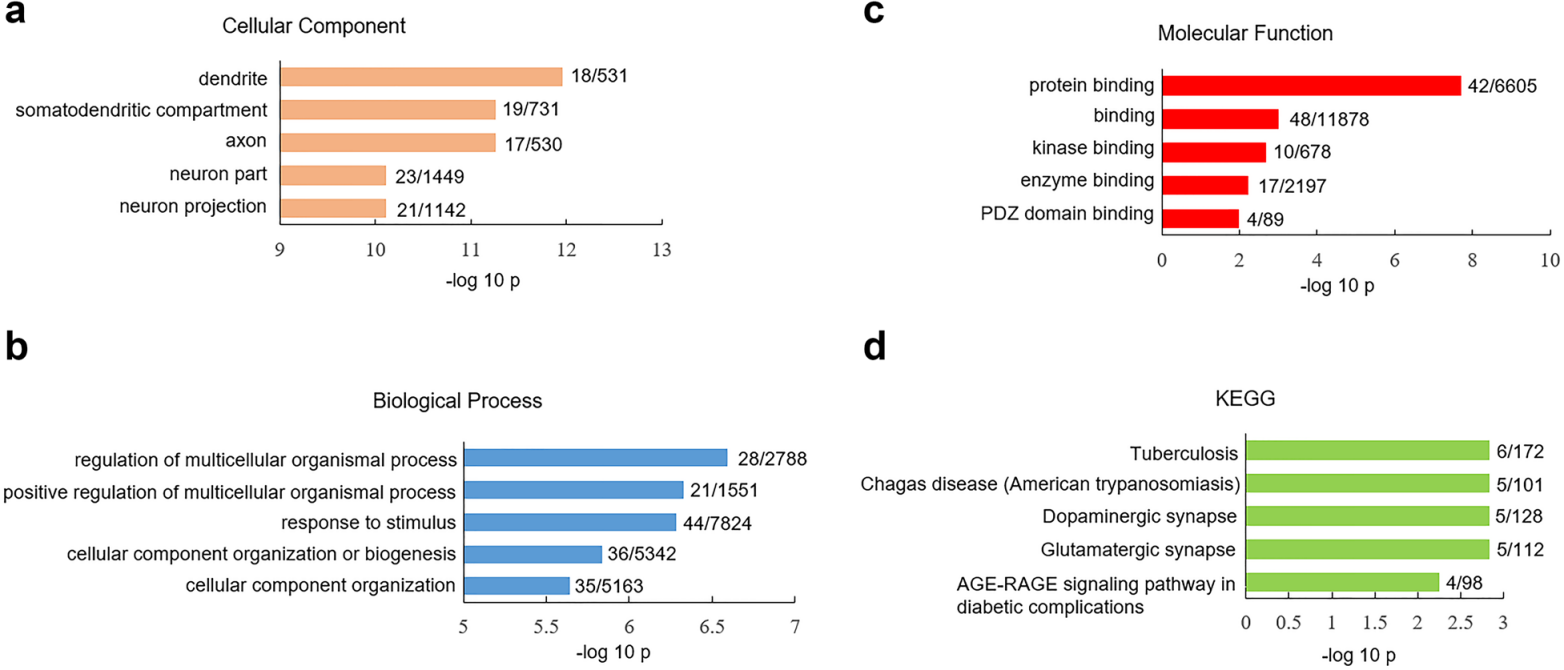
GO and KEGG analysis of differential expressed SRG in glutamatergic neurons between human versus mouse V1 cortex. Significantly overrepresented cellular component (**a**), biological process (**b**) and molecular function (**c**). **d** significantly overrepresented KEGG. The x-axis represents the value of −log_10_p, the y-axis indicates the item of GO or KEGG, the numbers after each bar indicate the list hits/pop hits

We next compare the expression of SRG in glutamatergic neurons between human versus mouse V1 cortex. The percentage of glutamatergic neurons expressing SRG was determined by the ratio of SRG-positive glutamatergic neurons to total glutamatergic neurons (Additional file 3: Table S3). Strikingly, the percentages of glutamatergic neurons expressing 54 out of 180 SRG were significantly lower in human V1 cortex than mouse V1 cortex (Additional file 3: Table S3). Having identified the subset of SRG that exhibited species difference in glutamatergic neurons, we sought to explore their biological characteristics using pathway analysis. GO-CC analysis of SRG with species difference in glutamatergic neurons revealed that they were enriched in the dendrite and axon (Fig. 3a). Species-different SRG in glutamatergic neurons showed unique enrichment in biological processes such as regulation of multicellular organismal process (p = 2.56 × 10^−7^) and response to stimulus (p = 4.72 × 10^−7^) (Fig. 3b). GO-MF analysis indicated that species-different SRG in glutamatergic neurons were highly presented in protein binding pathway (p = 2.01 × 10^−8^) (Fig. 3c). KEGG assay showed that species-different SRG in glutamatergic neurons were enriched in dopaminergic (p = 1.5 × 10^−3^) and glutamatergic synapse (p = 1.5 × 10^−3^) (Fig. 3d).

### Expression of SRG in GABAergic neurons from human versus mouse V1 cortex

**Fig. 4:**
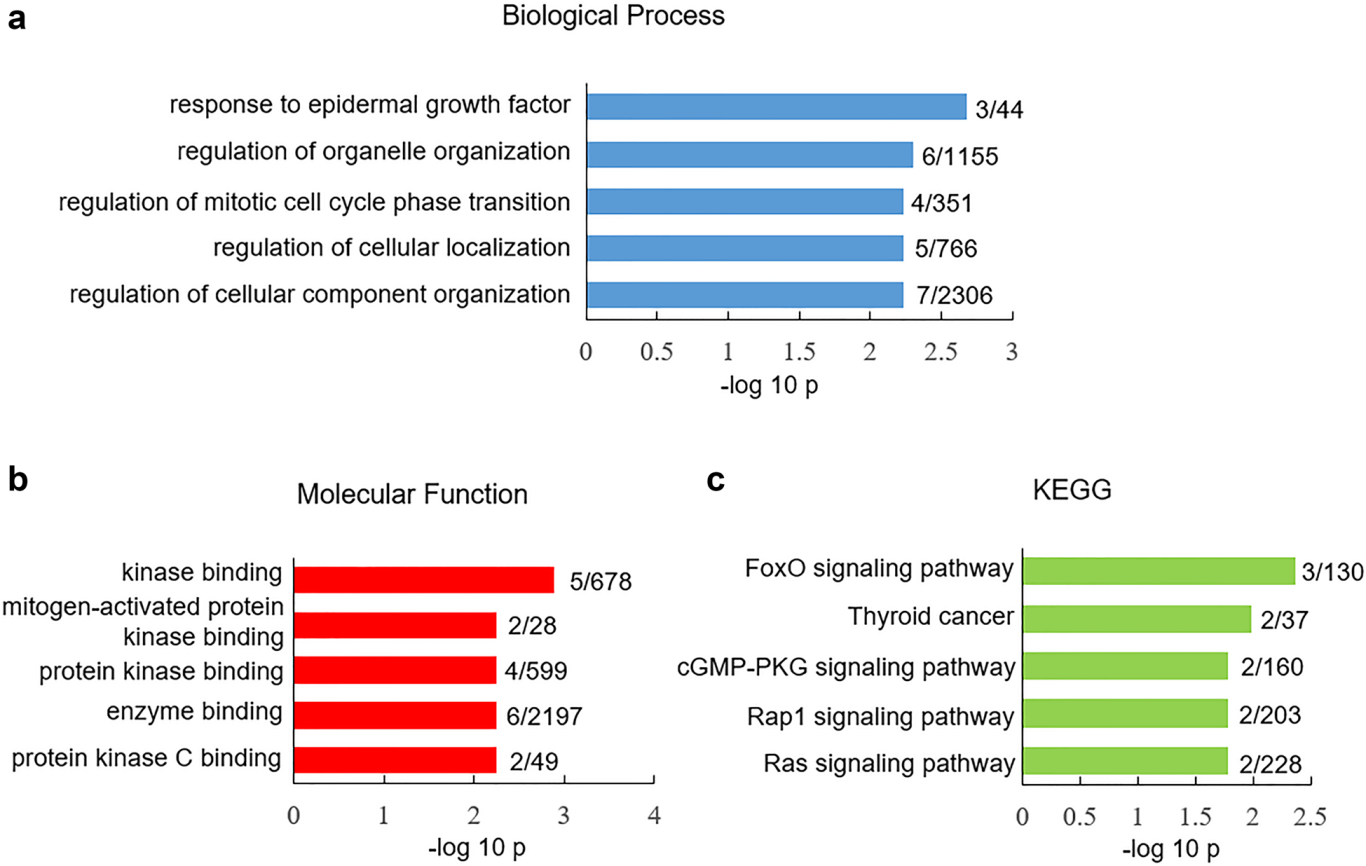
GO and KEGG analysis of differential expressed SRG in GABAergic neurons between human versus mouse V1 cortex. Significantly overrepresented biological process (**a**) and molecular function (**b**). **c** Significantly overrepresented KEGG. The x-axis represents the value of −log_10_p, the y-axis indicates the item of GO or KEGG, the numbers after each bar indicate the list hits/pop hits

In the following study we sought to compare the expression of SRG in GABAergic neurons between human versus mouse V1 cortex. The percentage of GABAergic neurons expressing SRG was determined by the ratio of SRG-positive GABAergic neurons to total GABAergic neurons (Additional file 4: Table S4). Unlike glutamatergic neurons, the percentages of GABAergic neurons expressing most SRG (170 out of 180) were similar between human versus mouse V1 cortex (Additional file 4: Table S4). Only ten SRG showed different expression in GABAergic neurons between human versus mouse V1 cortex (Additional file 4: Table S4). GO-CC analysis of species-different SRG in GABAergic neurons did not reveal any enrichment. Species-different SRG in GABAergic neurons showed unique enrichment in biological processes such as response to epidermal growth factor (p = 2.1 × 10^−3^) and regulation of organelle organization (p = 5 × 10^−3^) (Fig. 4a). GO-MF analysis indicated that species-different SRG in GABAergic neurons were highly presented in kinase binding pathway (p = 1.3 × 10^−3^) (Fig. 4b). KEGG assay showed that these species-different SRG were enriched in FoxO signaling pathway (p = 4.3 × 10^−3^) (Fig. 4c).

### Expression of SRG in non-neuronal cells from human versus mouse V1 cortex

We lastly compare the expression of SRG in non-neuronal cells between human versus mouse V1 cortex. The percentage of non-neuronal cells expressing SRG was determined by the ratio of SRG-positive non-neuronal cells to total non-neuronal cells (Additional file 5: Table S5). Different from neurons, the percentages of non-neuronal cells expressing all SRG were indistinguishable between human and mouse V1 cortex (Additional file 5: Table S5). These results indicated the species conservation of SRG expression in non-neuronal cells from human and mouse V1 cortex.

## Discussion

Here we analyzed the expression profile of 180 schizophrenia risk genes (SRG) in three cell types from human and mouse V1 cortex. We demonstrate that the majority of SRG had a consistent expression profile between mouse and human V1 cortex: 126 of 180 SRG are expressed with similar ratios in glutamatergic neurons, 170 out of 180 SRG are conserved in GABAergic neurons and all SRG are conserved in non-neuronal cells. These results support the rationality to use mouse models to study the function of SRG with similar expression pattern between human and mouse cortex. However, the gene expression pattern may different from brain regions [25]. It will be interesting to study whether the cellular expression pattern of SRG were conserved in other cortical brain regions such as prefrontal cortex and hippocampus. Such comparative studies may rely on the single-cell RNA sequence data from the matched brain regions which is not currently available.

For 30% SRG, however, their expression in glutamatergic neurons were significantly different between human and mouse V1 cortex. Only 10 SRG showed species difference in GABAergic neurons. Strikingly, the 10 SRG differently expressed in GABAergic neurons between human and mouse cortex also exhibit species difference in glutamatergic neurons. The different expression pattern of certain SRG between mouse and human V1 cortex may not due to the age because both adult mice and human cortical tissue were used for the assay in Allen Brain database. Near one-third SRG showed species difference in glutamatergic neurons, which may not result from the difference in overall gene transcription between human and mouse cortex because only a small proportion of genes exhibit human-specific cortex transcriptome signature [26, 27]. Although the reason for the species-different cellular expression pattern of certain SRG is not completely clear, we reason that it should be cautious to use mouse models to study the species-different SRG. Other models such as patient iPSC-derived neuronal culture or brain organoids may be alternative approaches to study the function of SRG.

## Conclusion

Here we compared the cellular expression pattern of SRG from matched brain regions of human versus mouse cortex. Our results indicate that GABAergic neurons are more conserved in the expression of SRG than glutamatergic neurons while the non-neuronal cells show the species conservation for the expression of all SRG. It should be cautious to use mouse models to study those SRG which show different cellular expression pattern between human and mouse cortex.

## Supplementary information

**Additional file 1: Table S1.** The number of SRG-positive and SRG-negative cells in the population of glutamatergic neuron, GABAergic neuron and non-neuronal cell.

**Additional file 2: Table S2.** The ratio of SRG-positive cells to total cells in human versus mouse V1 cortex. The κ^2^ and p value were shown for each SRG. The red color indicates significant difference between human and mouse.

**Additional file 3: Table S3.** The ratio of SRG-positive glutamatergic neurons to total glutamatergic neurons in human versus mouse V1 cortex. The κ^2^ and p value were shown for each SRG. The red color indicates significant difference between human and mouse.

**Additional file 4: Table S4.** The ratio of SRG-positive GABAergic neurons to total GABAergic neurons in human versus mouse V1 cortex. The κ^2^ and p value were shown for each SRG. The red color indicates significant difference between human and mouse.

**Additional file 5: Table S5.** The ratio of SRG-positive non-neuronal cell to total non-neuronal cell in human versus mouse V1 cortex. The κ^2^ and p value were shown for each SRG.

## Data Availability

All data generated and/or analyzed during this study are included in this published article and its supplementary information files.

## Abbreviations

SRG: schizophrenia risk gene
SZ: schizophrenia
GWAS: genome-wide association study
GO: gene ontology
CC: cellular compartment
BP: biological process
MF: molecular function
KEGG: Kyoto Encyclopedia of Gene and Genomes
iPSC: induced pluripotent stem cell
